# Supplementary material for: Kinematic Studies of the Go/No-Go Task as a Dynamic Sensorimotor Inhibition Task for Assessment of Motor and Executive Function in Stroke Patients: An Exploratory Study in a Neurotypical Sample
Source: Brain Sci. 2022 Nov 19;12(11):1581. doi: 10.3390/brainsci12111581 (PMC9688448; doi:10.3390/brainsci12111581)
Supplement: Supplementary file 1 [file brainsci-12-01581-s001.zip › brainsci-1915807-supplementary.pdf]

Supplementary materials

Supplementary Table S1: *Middle control key kinematic events from Study One, reported in terms of time, value and percent of trial at which the event occurred.*

Kinematic Event	Mean (SD)	Range
Reaction Time (ms)	240 (40)	180-320
Movement Time (ms)	620 (160)	330-940
Total Time (ms)	860 (190)	540-1200
Peak Acceleration Time (ms)	110 (30)	80-180
Peak Acceleration Value (mm/s^2)	10.31 (5.61)	5.04-31.99
Peak Acceleration % of trial	17.96 (4.37)	8.04-27.07
Peak Velocity Time (ms)	240 (50)	130-350
Peak Velocity Value (mm/s)	880 (250)	570-1630
Peak Velocity % of trial	40.33 (5.07)	29.59-46.47
Peak De-acceleration Time (ms)	420 (80)	230-570
Peak De-acceleration Value (mm/s^2)	-6.02 (3.26)	-14.80 - -2.55
Peak De-acceleration % of trial	69.95 (12.59)	47.04 – 90.27
Peak Grip Width Time (ms)	400 (80)	220-540
Peak Grip Width Value (mm)	114 (9.43)	96.33-129.05
Peak Grip Width % of trial	65.10 (7.94)	41.84-77.15

Supplementary Table S2. *Key kinematic events for Go trials for each Go/No-Go task in Study One, for the overall task and for each object interacted with, reported in terms of time, value and percent of trial at which the event occurred.*

Left Go/No-Go						
Kinematic event	Overall		Middle Object		Right Object	
	Mean (SD)	Range	Mean (SD)	Range	Mean (SD)	Range
Reaction Time (ms)	320 (90)	210-540	320 (80)	220-530	330 (100)	200-560
Movement Time (ms)	580 (150)	380-940	590 (160)	390-950	560 (150)	360-940
Total Time (ms)	900 (220)	630-1440	920 (220)	630-1370	890 (230)	620-1500
Peak Acceleration Time (ms)	120 (40)	70-200	120 (40)	70-200	120 (40)	70-220
Peak Acceleration Value (mm/s^2)	11.74 (5.29)	5-46	11.38 (5.33)	5.81-27.11	12.13 (5.31)	5.12-26.66
Peak Acceleration % of trial	20.62 (4.43)	9.32-31.03	19.81 (5.30)	8.82-29.32	21.58 (4.67)	9.82-32.74
Peak Velocity Time (ms)	240 (60)	140-350	240 (70)	140-350	230 (60)	130-360
Peak Velocity Value (mm/s)	990 (290)	570-1790	960 (290)	550-1900	1020 (290)	590-1670
Peak Velocity % of trial	41.62 (4.67)	32.66-48.94	41.47 (4.87)	31.62-48.39	41.84 (4.70)	32.11-49.77
Peak De-acceleration Time (ms)	390 (100)	180-590	420 (110)	180-630	350 (110)	190-560
Peak De-acceleration Value (mm/s^2)	-7.97 (5.41)	-27.76- -2.92	-7.32 (5.37)	-27.44- -2.34	-8.67 (5.55)	-28.08- -3.27
Peak De-acceleration % of trial	67.87 (12.23)	43.65–92.56	72.05 (14.29)	40.22-92.76	62.77 (12.55)	45.16-92.36
Peak Grip Width Time (ms)	380 (90)	270-580	390 (90)	270-590	370 (90)	260-570
Peak Grip Width Value (mm)	120.83 (11.59)	99.28-145	114.95 (9.24)	96.36-134.52	126.54 (14.95)	101.81-156.50
Peak Grip Width % of trial	66.63 (6.25)	52.07-76.42	66.36 (6.81)	48.67-77.75	66.90 (6.36)	55.47-78.42
Middle Go/No-Go						

Kinematic event	Overall		Left Object		Right Object	
	Mean (SD)	Range	Mean (SD)	Range	Mean (SD)	Range
Reaction Time (ms)	330 (80)	230-530	320 (80)	240-510	330 (80)	220-550
Movement Time (ms)	590 (170)	370-1000	530 (170)	390-1030	570 (170)	340-970
Total Time (ms)	920 (230)	650-1430	930 (220)	670-1430	910 (230)	610-1460
Peak Acceleration Time (ms)	120 (40)	70-230	110 (40)	70-240	130 (50)	70-230
Peak Acceleration Value (mm/s^2)	11.92 (5.59)	4.86-29.19	11.05 (5.07)	4.77-26.54	12.88 (6.20)	4.95-31.66
Peak Acceleration % of trial	20.70 (4.51)	10.18-29.31	19.31 (4.68)	9.87-27.79	22.17 (5.32)	10.48-30.83
Peak Velocity Time (ms)	250 (70)	130-390	260 (70)	150-380	230 (70)	120-390
Peak Velocity Value (mm/s)	1030 (290)	560-1800	1040 (310)	560-2020	1020 (280)	560-1600
Peak Velocity % of trial	42.47 (4.82)	28.17-48.99	43.18 (4.63)	29.86-49.65	41.30 (5.42)	26.59-49.66
Peak De-acceleration Time (ms)	400 (110)	190-640	430 (120)	200-720	360 (110)	190-570
Peak De-acceleration Value (mm/s^2)	-7.84 (5.02)	-27.15- -2.55	-7.27 (5.37)	-28.93- -2.38	-8.85 (5.44)	-25.49- -2.71
Peak De-acceleration % of trial	68.71 (10.56)	41.38-88.20	73.26 (13.20)	39.63-90.37	63.23 (11.15)	43.01-91.16
Peak Grip Width Time (ms)	390 (100)	260-620	410 (110)	270-640	380 (100)	250-610
Peak Grip Width Value (mm)	120.22 (13.15)	99.14-145.88	113.55 (11.12)	94.97-134.98	127.33 (16.21)	103.04-160.48
Peak Grip Width % of trial	67.45 (5.27)	55.30-75.70	67.93 (5.59)	54.95-77.92	66.94 (5.90)	55.29-75.42

Right Go/No-Go

Kinematic event	Overall		Left Object		Middle Object	
	Mean (SD)	Range	Mean (SD)	Range	Mean (SD)	Range
Reaction Time (ms)	320 (70)	210-570	320 (70)	230-590	320 (80)	200-550
Movement Time (ms)	600 (140)	400-980	600 (140)	400-960	600 (140)	400-990

Total Time (ms)	910 (180)	660-1380	910 (170)	650-1320	910 (190)	660-1420
Peak Acceleration Time (ms)	120 (40)	80-200	110 (30)	70-180	120 (50)	60-220
Peak Acceleration Value (mm/s^2)	10.34 (3.82)	5.97-18.41	10.45 (4.02)	5.95-18.82	10.24 (3.66)	5.99-18.03
Peak Acceleration % of trial	19.47 (4.79)	11.11-27.33	18.40 (4.55)	10.46-26.69	20.52 (5.87)	10.19-29.62
Peak Velocity Time (ms)	250 (50)	170-360	260 (50)	180-360	250 (50)	170-360
Peak Velocity Value (mm/s)	960 (200)	610-1420	1000 (220)	640-1480	910 (190)	580-1360
Peak Velocity % of trial	43.41 (4.30)	35.98-49.06	44.29 (4.12)	36.54-49.79	42.53 (4.77)	32.12-48.65
Peak De-acceleration Time (ms)	420 (80)	320-610	430 (80)	340-620	420 (80)	290-600
Peak De-acceleration Value (mm/s^2)	-5.98 (2.31)	-12.01- -2.79	-6.06 (2.51)	-12.28- -2.71	-5.92 (2.14)	-11.73- -2.86
Peak De-acceleration % of trial	72.98 (11.59)	52.19-92.75	73.81 (11.57)	54.71-91.37	72.06 (12.55)	49.47-95.65
Peak Grip Width Time (ms)	400 (90)	270-650	400 (90)	270-650	390 (90)	270-640
Peak Grip Width Value (mm)	113.01 (9.45)	94.68-130.22	112.70 (9.81)	93.06-131.32	113.32 (9.29)	95.23-129.46
Peak Grip Width % of trial	67.29 (6.41)	53.45-76.90	68.20 (6.39)	53.01-78.23	66.40 (6.73)	53.76-78

Supplementary Table S3. *The timing of key kinematic events captured in each of the Go/No-Go tasks in Study One, including average number of occurrences per task and the mean time at which they occurred. Due to small number of trials in Wrist Movement where movement stop could be captured, the time it took to stop is reported instead of chronological timepoint at which the stop occurred.*

Kinematic event	Left Go/No-go			Middle Go/No-go			Right Go/No-go		
	N	Mean (SD)	Range	N	Mean (SD)	Range	N	Mean (SD)	Range
Wrist Movement									
Number of trials	25	2.24 (1.79)	0-6	25	2.28 (1.95)	0-7	23	2.30 (2.69)	0-11
Reaction Time (ms)	20	760 (490)	270-2010	23	760 (490)	230—1810	15	760 (430)	210-1480
Time to Stop (ms)	10	118 (90)	19-356	15	121 (70)	52-326	5	110 (70)	64-228
Miss Reach									
Number of trials	25	1.30 (1.61)	0-6	25	1.36 (1.29)	0-4	23	1.04 (1.07)	0-3
Reaction Time (ms)	14	420 (250)	230-1250	17	440 (330)	210-1560	14	370 (120)	230-650
Peak Velocity Time (ms)	14	620 (510)	370-2360	17	610 (330)	270-1640	14	520 (210)	280-950
Stop Time (ms)	14	780 (510)	490-2480	15	670 (300)	470-1700	12	660 (230)	380-1180

Supplementary Table S4: Non-parametric correlation matrix (Kendall's Tau B) For the AQ and AQ Subscales, DASS-21 and DASS-21 Subscales, Ravens, SLURP and Average Go/No-Go Responses

	**AQ5**	**AQsocial**	**AQattswit**	**AQattdet**	**AQcomm**	**AQimag**
AQsocial	τb(28)=0.435, p=.003	—				
AQattswit	τb(28)=0.319, p=.029	τb(28)=0.111, p=.474	—			
AQattdet	τb(28)=0.496, P<.001	τb(28)=0.057, p=.707	τb(28)=0.177, p=.236	—		
AQcomm	τb(28)=0.419, p=.004	τb(28)=0.166, p=.288	τb(28)=-0.051, p=.739	τb(28)=0.121, p=.421	—	
AQimag	τb(28)=0.351, p=.016	τb(28)=0.234, p=.130	τb(28)=-0.138, p=.364	τb(28)=0.064, p=.668	τb(28)=0.208, p=.176	—
RavenAv	τb(28)=-0.222, p=.104	τb(28)=-0.102, p=.483	τb(22)=-0.21, p=.140	τb(28)=-0.173, p=.215	τb(28)=0.053, p=.714	τb(28)=-0.147, p=.303
DASS21	τb(28)=0.165, p=.233	τb(28)=-0.191, p=.193	τb(28)=0.135, p=.352	τb(28)=0.202, p=.154	τb(28)=0.254, p=.082	τb(28)=0.035, p=.808
DASS_D	τb(28)=0.18, p=.205	τb(28)=-0.076, p=.616	τb(28)=0.064, p=.667	τb(28)=0.095, p=.517	τb(28)=0.314, p=.037	τb(28)=0.1, p=.500
DASS_A	τb(28)=0.324, p=.025	τb(28)=-0.101, p=.513	τb(28)=0.125, p=.408	τb(28)=0.322, p=.030	τb(28)=0.214, p=.162	τb(28)=0.25, p=.098
DASS_S	τb(28)=-0.025, p=.857	τb(28)=-0.337, p=.024	τb(28)=0.143, p=.329	τb(28)=0.127, p=.376	τb(28)=0.073, p=.623	τb(28)=-0.089, p=.543
AV RT MR	τb(28)=0.101, p=.501	τb(24)=-0.025, p=.876	τb(24)=0.19, p=.222	τb(24)=-0.069, p=.650	τb(24)=0.214, p=.174	τb(24)=0.016, p=.919
Tot N MR	τb(28)=0.012, p=.936	τb(28)=-0.003, p=.983	τb(28)=0.227, p=.129	τb(28)=-0.173, p=.239	τb(28)=0.238, p=.116	τb(28)=-0.153, p=.306
AV RT WM	τb(24)=-0.216, p=.148	τb(24)=-0.128, p=.419	τb(24)=-0.389, p=.012	τb(24)=-0.107, p=.482	τb(24)=0.032, p=.839	τb(24)=0.074, p=.631
Tot N WM	τb(28)=-0.116, p=.412	τb(28)=-0.196, p=.191	τb(28)=-0.087, p=.555	τb(28)=-0.125, p=.386	τb(28)=0.034, p=.821	τb(28)=0.021, p=.887

AV RT GO	τb(28)=-0.125, p=.361	τb(28)=-0.012, p=.934	τb(28)=-0.118, p=.408	τb(28)=-0.062, p=.660	τb(28)=-0.035, p=.807	τb(28)=-0.118, p=.408
STotMT	τb(22)=0.182, p=.245	τb(22)=0.167, p=.321	τb(22)=0.215, p=.185	τb(22)=0.381, p=.017	τb(22)=-0.204, p=.220	τb(22)=-0.085, p=.603
STotME	τb(22)=0.036, p=.821	τb(22)=0.035, p=.833	τb(22)=0.155, p=.342	τb(22)=-0.092, p=.567	τb(22)=0.131, p=.430	τb(22)=0.203, p=.214
SEasMT	τb(22)=0.325, p=.039	τb(22)=0.197, p=.241	τb(22)=0.327, p=.044	τb(22)=0.446, p=.005	τb(22)=0.165, p=.320	τb(22)=-0.076, p=.644
SEasME	τb(22)=0.188, p=.233	τb(22)=0.015, p=.928	τb(22)=0.225, p=.167	τb(22)=0.06, p=.710	τb(22)=0.312, p=.061	τb(22)=0.128, p=.435
SMedMT	τb(22)=0.147, p=.350	τb(22)=0.167, p=.321	τb(22)=0.168, p=.300	τb(22)=0.418, p=.009	τb(22)=-0.233, p=.161	τb(22)=-0.057, p=.729
SMedME	τb(22)=0.009, p=.955	τb(22)=0.137, p=.417	τb(22)=-0.009, p=.954	τb(22)=-0.148, p=.359	τb(22)=-0.073, p=.661	τb(22)=0.261, p=.112
SHarMT	τb(22)=0.111, p=.429	τb(22)=0.167, p=.321	τb(22)=0.206, p=.205	τb(22)=0.262, p=.103	τb(22)=-0.32, p=.054	τb(22)=-0.028, p=.862
SHarME	τb(22)=-0.144, p=.363	τb(22)=0.072, p=.673	τb(22)=-0.014, p=.931	τb(22)=-0.232, p=.152	τb(22)=-0.083, p=.619	τb(22)=0.267, p=.105

	**RavenAv**	**DASS21**	**DASS_D**	**DASS_A**	**DASS_S**	**AV RT MR**
AQsocial						
AQattswit						
AQattdet						
AQcomm						
AQimag						
RavenAv	—					
DASS21	τb(28)=-0.188, p=.166	—				
DASS_D	τb(28)=-0.187, p=.180	τb(28)=0.718, p<.001	—			
DASS_A	τb(28)=-0.161, p=.257	τb(28)=0.541, p<.001	τb(22)=0.41, p=.006	—		
DASS_S	τb(28)=-0.2, p=.147	τb(28)=0.743, p.001	τb(28)=0.506, p<.001	τb(28)=0.312, p=.032	—	
AV RT MR	τb(24)=-0.047, p=.747	τb(24)=0.17, p=.252	τb(24)=0.167, p=.270	τb(24)=-0.031, p=.839	τb(24)=0.223, p=.140	—

Tot N MR	τb(28)=-0.011, p=.936	τb(28)=0.083, p=.560	τb(28)=0.089, p=.542	τb(28)=-0.049, p=.742	τb(28)=0.061, p=.672	τb(24)=0.137, p=.375
AV RT WM	τb(24)=0.109, p=.476	τb(24)=0.044, p=.765	τb(24)=0.08, p=.598	τb(24)=-0.085, p=.578	τb(24)=0.116, p=.438	τb(21)=0.229, p=.158
Tot N WM	τb(28)=-0.049, p=.720	τb(28)=-0.073, p=.604	τb(28)=-0.148, p=.303	τb(28)=-0.143, p=.327	τb(28)=-0.003, p=.984	τb(24)=0.211, p=.161
AV RT GO	τb(28)=0.185, p=.174	τb(28)=-0.253, p=.063	τb(28)=-0.21, p=.133	τb(28)=-0.052, p=.715	τb(28)=-0.244, p=.077	τb(24)=-0.062, p=.673
STotMT	τb(22)=-0.139, p=.367	τb(22)=-0.176, p=.258	τb(22)=-0.31, p=.052	τb(22)=0.019, p=.907	τb(22)=-0.148, p=.348	τb(19)=-0.199, p=.234
STotME	τb(22)=-0.022, p=.888	τb(22)=0.198, p=.203	τb(22)=0.096, p=.548	τb(22)=0.138, p=.398	τb(22)=0.234, p=.139	τb(19)=0.124, p=.462
SEasMT	τb(22)=-0.113, p=.463	τb(22)=-0.062, p=.692	τb(22)=-0.128, p=.423	τb(22)=0.028, p=.861	τb(22)=-0.076, p=.629	τb(19)=-0.035, p=.834
SEasME	τb(22)=-0.087, p=.572	τb(22)=0.415, p=.008	τb(22)=0.238, p=.136	τb(22)=0.267, p=.103	τb(22)=0.383, p=.016	τb(19)=0.189, p=.262
SMedMT	τb(22)=-0.182, p=.236	τb(22)=-0.167, p=.282	τb(22)=-0.301, p=.059	τb(22)=0.019, p=.907	τb(22)=-0.175, p=.268	τb(19)=-0.235, p=.161
SMedME	τb(22)=0.066, p=.672	τb(22)=0.066, p=.671	τb(22)=0.078, p=.626	τb(22)=0.033, p=.838	τb(22)=0.036, p=.820	τb(19)=0.083, p=.624
SHarMT	τb(22)=-0.095, p=.535	τb(22)=-0.317, p=.042	τb(22)=-0.429, p=.007	τb(22)=-0.133, p=.415	τb(22)=-0.265, p=.094	τb(19)=-0.246, p=.141
SHarME	τb(22)=0.11, p=.480	τb(22)=-0.044, p=.777	τb(22)=-0.097, p=.547	τb(22)=-0.106, p=.521	τb(22)=0.032, p=.842	τb(19)=-0.006, p=.972

	Tot N MR	AV RT WM	Tot N WM	AV RT GO	STotMT	STotME
AQsocial						
AQattswit						
AQattdet						
AQcomm						
AQimag						
RavenAv						
DASS21						
DASS_D						

DASS_A						
DASS_S						
AV RT MR						
Tot N MR	—					
AV RT WM	τb(24)=-0.119, p=.436	—				
Tot N WM	τb(28)=0.07, p=.628	τb(24)=0.217, p=.153	—			
AV RT GO	τb(28)=-0.163, p=.245	τb(24)=0.065, p=.677	τb(28)=-0.022, p=.873	—		
STotMT	τb(22)=-0.174, p=.277	τb(19)=-0.246, p=.141	τb(22)=-0.14, p=.377	τb(22)=0.286, p=.063	—	
STotME	τb(22)=0.078, p=.627	τb(19)=-0.094, p=.575	τb(22)=-0.2, p=.209	τb(22)=0.039, p=.800	τb(22)=0.074, p=.631	—
SEasMT	τb(22)=0.018, p=.909	τb(19)=-0.246, p=.141	τb(22)=-0.086, p=.588	τb(22)=0.208, p=.176	τb(22)=0.574, p<.001	τb(22)=-0.013, p=.933
SEasME	τb(22)=0.17, p=.290	τb(19)=-0.182, p=.278	τb(22)=-0.096, p=.549	τb(22)=-0.044, p=.778	τb(22)=-0.122, p=.429	τb(22)=0.6, p<.001
SMedMT	τb(22)=-0.202, p=.208	τb(19)=-0.176, p=.294	τb(22)=-0.14, p=.377	τb(22)=0.243, p=.114	τb(22)=0.852, p<.001	τb(22)=0.013, p=.933
SMedME	τb(22)=-0.088, p=.586	τb(19)=-0.006, p=.927	τb(22)=-0.274, p=.087	τb(22)=-0.048, p=.756	τb(22)=0.022, p=.888	τb(22)=0.614, p<.001
SHarMT	τb(22)=-0.174, p=.277	τb(19)-0.164, p=.327	τb(22)=-0.095, p=.549	τb(22)=0.156, p=.310	τb(22)=0.73, p<.001	τb(22)=0.031, p=.843
SHarME	τb(22)=-0.014, p=.931	τb(19)=0.024, p=.889	τb(22)=-0.064, p=.689	τb(22)=-0.022, p=.888	τb(22)=0.004, p=.977	τb(22)=0.678, p<.001

	**Tot N MR**	**AV RT WM**	**Tot N WM**	**AV RT GO**	**STotMT**	**STotME**
AQsocial						
AQattswit						
AQattdet						
AQcomm						

AQimag						
RavenAv						
DASS21						
DASS_D						
DASS_A						
DASS_S						
AV RT MR						
Tot N MR	—					
AV RT WM	τb(24)=-0.119, p=.436	—				
Tot N WM	τb(28)=0.07, p=.628	τb(24)=0.217, p=.153	—			
AV RT GO	τb(28)=-0.163, p=.245	τb(24)=0.065, p=.677	τb(28)=-0.022, p=.873	—		
STotMT	τb(22)=-0.174, p=.277	τb(19)=-0.246, p=.141	τb(22)=-0.14, p=.377	τb(22)=0.286, p=.063	—	
STotME	τb(22)=0.078, p=.627	τb(19)=-0.094, p=.575	τb(22)=-0.2, p=.209	τb(22)=0.039, p=.800	τb(22)=0.074, p=.631	—
SEasMT	τb(22)=0.018, p=.909	τb(19)=-0.246, p=.141	τb(22)=-0.086, p=.588	τb(22)=0.208, p=.176	τb(22)=0.574, p<.001	τb(22)=-0.013, p=.933
SEasME	τb(22)=0.17, p=.290	τb(19)=-0.182, p=.278	τb(22)=-0.096, p=.549	τb(22)=-0.044, p=.778	τb(22)=-0.122, p=.429	τb(22)=0.6, p<.001
SMedMT	τb(22)=-0.202, p=.208	τb(19)=-0.176, p=.294	τb(22)=-0.14, p=.377	τb(22)=0.243, p=.114	τb(22)=0.852, p<.001	τb(22)=0.013, p=.933
SMedME	τb(22)=-0.088, p=.586	τb(19)=-0.006, p=.927	τb(22)=-0.274, p=.087	τb(22)=-0.048, p=.756	τb(22)=0.022, p=.888	τb(22)=0.614, p<.001
SHarMT	τb(22)=-0.174, p=.277	τb(19)-0.164, p=.327	τb(22)=-0.095, p=.549	τb(22)=0.156, p=.310	τb(22)=0.73, p<.001	τb(22)=0.031, p=.843
SHarME	τb(22)=-0.014, p=.931	τb(19)=0.024, p=.889	τb(22)=-0.064, p=.689	τb(22)=-0.022, p=.888	τb(22)=0.004, p=.977	τb(22)=0.678, p<.001

	**SEasMT**	**SEasME**	**SMedMT**	**SMedME**	**SHarMT**

AQsocial					
AQattswit					
AQattdet					
AQcomm					
AQimag					
RavenAv					
DASS21					
DASS_D					
DASS_A					
DASS_S					
AV RT MR					
Tot N MR					
AV RT WM					
Tot N WM					
AV RT GO					
STotMT					
STotME					
SEasMT	—				
SEasME	τb(22)=0.061, p=.693	—			
SMedMT	τb(22)=0.443, p=.004	τb(22)=-0.236, p=.127	—		
SMedME	τb(22)=-0.249, p=.107	τb(22)=0.207, p=.184	τb(22)=0.013, p=.932	—	
SHarMT	τb(22)=0.339, p=.028	τb(22)=-0.218, p=.158	τb(22)=0.67, p<.001	τb(22)=0.109, p=.48-	—
SHarME	τb(22)=-0.101, p=.515	τb(22)=0.331, p=.034	τb(22)=-0.04, p=.799	τb(22)=0.522, p<.001	τb(22)=0.11, p=.480
	* p < .05,	** p < .01,	*** p < .001		
